# Changes in both top-down and bottom-up effective connectivity drive visual hallucinations in Parkinson’s disease

**DOI:** 10.1093/braincomms/fcac329

**Published:** 2022-12-14

**Authors:** George E C Thomas, Peter Zeidman, Tajwar Sultana, Angeliki Zarkali, Adeel Razi, Rimona S Weil

**Affiliations:** Dementia Research Centre, UCL Institute of Neurology, WC1N 3AR London, UK; Wellcome Centre for Human Neuroimaging, UCL Institute of Neurology, WC1N 3AR London, UK; Department of Computer and Information Systems Engineering, NED University of Engineering & Technology, Karachi 75270, Pakistan; Department of Biomedical Engineering, NED University of Engineering & Technology, Karachi 74800, Pakistan; Neurocomputation Laboratory, NCAI Computer and Information Systems Department, NED University of Engineering and Technology, Karachi 75270, Pakistan; Dementia Research Centre, UCL Institute of Neurology, WC1N 3AR London, UK; Wellcome Centre for Human Neuroimaging, UCL Institute of Neurology, WC1N 3AR London, UK; Turner Institute for Brain and Mental Health, School of Psychological Sciences, Monash University, Clayton, VIC 3800, Australia; CIFAR Azrieli Global Scholars Program, CIFAR, Toronto, ON M5G 1M1, Canada; Dementia Research Centre, UCL Institute of Neurology, WC1N 3AR London, UK; Wellcome Centre for Human Neuroimaging, UCL Institute of Neurology, WC1N 3AR London, UK; Movement Disorders Consortium, UCL, London, UK

**Keywords:** spectral dynamic causal modelling, resting-state functional MRI, Parkinson’s disease, visual hallucinations, effective connectivity

## Abstract

Visual hallucinations are common in Parkinson’s disease and are associated with a poorer quality of life and a higher risk of dementia. An important and influential model that is widely accepted as an explanation for the mechanism of visual hallucinations in Parkinson’s disease and other Lewy body diseases is that these arise due to aberrant hierarchical processing, with impaired bottom-up integration of sensory information and overweighting of top-down perceptual priors within the visual system. This hypothesis has been driven by behavioural data and supported indirectly by observations derived from regional activation and correlational measures using neuroimaging. However, until now, there was no evidence from neuroimaging for differences in causal influences between brain regions measured in patients with Parkinson’s hallucinations. This is in part because previous resting-state studies focused on functional connectivity, which is inherently undirected in nature and cannot test hypotheses about the directionality of connectivity. Spectral dynamic causal modelling is a Bayesian framework that allows the inference of effective connectivity—defined as the directed (causal) influence that one region exerts on another region—from resting-state functional MRI data. In the current study, we utilize spectral dynamic causal modelling to estimate effective connectivity within the resting-state visual network in our cohort of 15 Parkinson’s disease visual hallucinators and 75 Parkinson’s disease non-visual hallucinators. We find that visual hallucinators display decreased bottom-up effective connectivity from the lateral geniculate nucleus to primary visual cortex and increased top-down effective connectivity from the left prefrontal cortex to primary visual cortex and the medial thalamus, as compared with non-visual hallucinators. Importantly, we find that the pattern of effective connectivity is predictive of the presence of visual hallucinations and associated with their severity within the hallucinating group. This is the first study to provide evidence, using resting-state effective connectivity, to support a model of aberrant hierarchical predictive processing as the mechanism for visual hallucinations in Parkinson’s disease.

## Teaser

Thomas *et al*. use dynamic causal modelling applied to functional MRI to show that both decreased bottom-up connectivity and increased top-down connectivity within the visual network contribute to visual hallucinations in Parkinson’s disease and that this pattern of directional connectivity is associated with the clinical severity of hallucinations.

## Graphical Abstract

**Graphical abstract:**
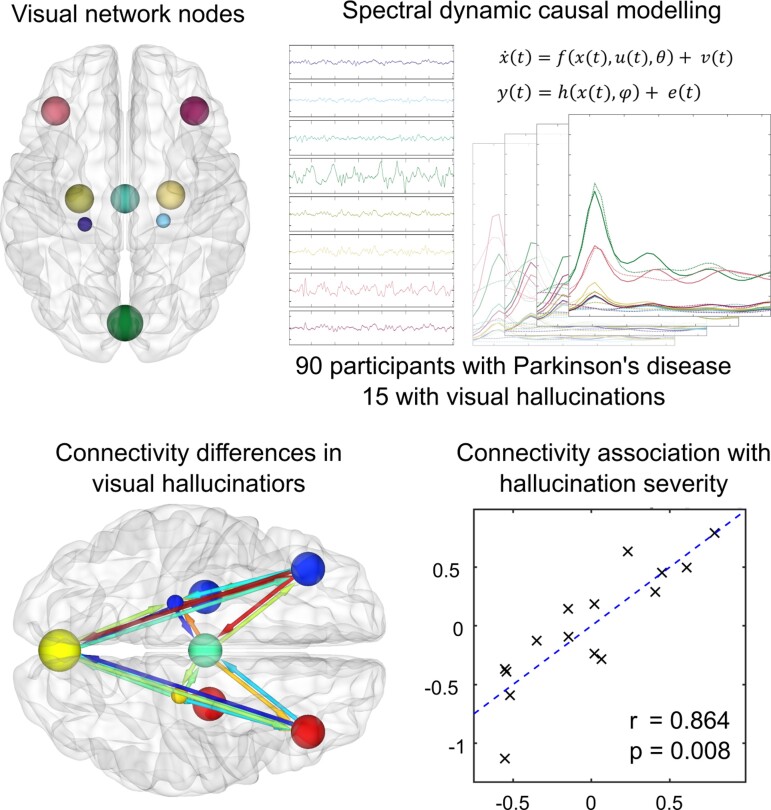

## Introduction

Visual hallucinations (VHs) are common in Parkinson’s disease, affecting up to 70% of patients.^1^ Parkinson’s disease patients who suffer from VH have a poorer quality of life,^2^ have an increased rate of mortality^3^ and are more likely to require nursing home care^4^ and to develop subsequent dementia.^5^ Until recently, models for how VH are generated within the brain remained somewhat limited and disparate, despite the high prevalence of VH and the clear burden they have on patients and their carers.^6^

However, over the last few years, lines of evidence have converged on a predictive-coding framework that suggests the occurrence of VH relates to altered integration of sensory input (bottom-up information) and prior knowledge (top-down information, or a ‘world model’) within the visual system.^7,8^ Patients with Parkinson’s disease frequently have deficits in visual processing,^6,9-12^ with potentially greater deficits in higher order visual processing in Parkinson’s patients who hallucinate compared with those that do not.^13^ Behavioural evidence has pointed specifically to impaired bottom-up accumulation of sensory evidence in VHs as compared with non-VHs,^14^ and patients with Lewy body related VH have been shown to rely more strongly on top-down perceptual priors.^15^ Notably, dorsolateral regions of the prefrontal cortex (PFC) are thought to play a key role in controlling top-down causal prediction error signalling.^16,17^

Several key brain regions have been implicated in Parkinson’s hallucinations. The thalamus is expected to be important in this framework as the synchronized functioning of these systems during normal visual perception relies strongly on thalamocortical connections.^18,19^ Previous work has shown that Parkinson’s disease hallucinators have reduced fibre cross section in white matter tracts connected to the medial thalamus early in the disease course, with this spreading to almost the whole thalamus in later disease.^20^ Additionally, lesion network mapping has revealed that a network centred on the lateral geniculate nucleus (LGN) is consistently affected in studies of Parkinson’s hallucinations^21^ and in patients with brain lesions causing VHs.^22^

Evidence also points towards the hippocampus as being important for application of perceptual priors due to its role in the encoding and retrieval of event-related memories^23^ as well as the integration of spatial and non-spatial contextual information.^24^ In Parkinson’s disease, VH has been linked to a higher burden of Lewy-related pathology in the medial temporal lobes.^13,25^ Alterations in functional connectivity to the hippocampus are seen in Parkinson’s hallucinators, with increased functional connectivity to the default mode and frontal regions and decreased functional connectivity with the occipital cortex.^26^

Primary visual cortex and frontal regions have been implicated in numerous functional MRI studies of Parkinson’s hallucinators.^27^ During exposure to simple visual stimuli, decreased activation in the primary visual and occipital cortex,^28-30^ has frequently been observed alongside increased activation in frontal^28,30^ and visual association regions^29^ in VHs. Exposure to complex visual stimuli in Parkinson’s disease with VH is associated with decreased frontal cortical activation^31,32^ and with altered functional connectivity in and between the attentional and default mode networks.^33^

Altered functional connectivity has also been reported in resting-state analyses comparing Parkinson’s disease VHs with non-VHs.^27^ Studies looking at the default mode network have found mostly increased functional connectivity in VHs compared with non-VHs, including in frontal-parietal regions.^34,35^ Increased connectivity within and between the default mode and attentional networks has also been found to correlate with performance on a complex visual task.^36^ Hallucination severity is associated with strengthened stability of the default mode network,^37^ and greater mind wandering in VHs linked with increased functional connectivity between primary visual cortex and the dorsal default mode network.^38^

Outside of the default mode network, increased occipital functional connectivity has been described with frontal and cortico-striatal regions^39^ and decreased functional connectivity has been reported between the posterior cingulate cortex and parietal, temporal and occipital regions^40^ in Parkinson’s disease with VH compared with Parkinson’s disease without VH.

Whilst these studies confirm altered connectivity and activation within visual network regions relating to VH in Parkinson’s disease, these studies only examine differences in functional connectivity, which is a measure of the statistical dependencies or correlations between neuroimaging time series. The correlational, undirected nature of functional connectivity precludes any assessment of the *causal* influences of distinct brain regions and cannot provide a mechanistic explanation of the neural mechanisms of Parkinson’s hallucinations based on hierarchical predictive processing, which requires information on directionality to address the question of the relative importance of bottom-up and top-down signalling.

Here, we employ dynamic causal modelling—a Bayesian framework that allows us to infer effective connectivity, which is designated as the directed (causal) influences among brain regions.^41^ A dynamic causal model (DCM) is defined by a forward model that generates neuroimaging time series based on the underlying causes, controlled by the model parameters. These parameters represent quantities such as connection strengths between regions and fall into three categories: neuronal parameters, haemodynamic parameters and parameters due to noise or measurement error.^42^ Once a DCM is specified, data can be simulated under different variations of the model (for example, with different connection strengths or architectures) to determine which model best characterizes the observed data. Hypotheses within or between subjects can then be tested by comparing the evidence for different models.

When modelling resting-state functional MRI data, the DCM methodology can become computationally intensive, due to the need to estimate random fluctuations in neuronal states.^43^ Spectral DCM^41^ has the advantage over conventional DCM when applied to resting-state data, in that, rather than modelling time-varying fluctuations in neuronal states, it models their second-order statistics or cross-spectra. This essentially models the dynamics of different brain regions in the frequency domain rather than the time domain. In doing so, it eliminates the need to estimate random neuronal fluctuations and is therefore more computationally efficient.^44^ Additionally, it provides a reliable estimation of between region influences as well as enables the accurate detection of group differences in effective connectivity.

In the current study, we used spectral DCM to estimate effective connectivity within a set of pre-defined visual brain regions in Parkinson’s disease patients with and without VHs. We investigate the importance of top-down and bottom-up connectivity within the visual network in explaining the differences between these groups, as well as the relative contributions of different regions and hemispheric connections to provide information about the architecture of this connectivity. The hierarchical nature of the visual network allows for the formation of strong hypotheses about inter-regional connectivity within the context of predictive processing, particularly when relating to symptoms that would be expected to arise due to aberrant processing of visual information.

We hypothesized that the presence of VH would be associated with both reduced bottom-up and increased top-down connectivity within the visual network and that the severity of hallucinations would be associated with the pattern of changes in connectivity.

## Materials and Methods

### Participants

We recruited 100 patients with Parkinson’s disease to our UK centre from affiliated clinics. Inclusion criteria were a diagnosis of Parkinson’s disease within 10 years, no history of traumatic brain injury or other psychiatric or neurological disorders, no contraindication to MRI and no ophthalmic disease sufficient to significantly impair visual acuity. Patients with Parkinson’s disease satisfied the Queen Square Brain Bank Criteria for Parkinson’s disease^45^ and did not suffer from dementia. The study was approved by the Queen Square Research Ethics Committee and all participants provided written, informed consent.

All participants underwent detailed clinical assessments. Motor function was assessed using the Movement Disorders Society Unified Parkinson's Disease Rating Scale (MDS-UPDRS) part III.^46^ Cognition was tested using the Montreal Cognitive Assessment (MoCA).^47^ LogMAR was used to assess visual acuity.^48^ Sniffin’ Sticks were used to assess smell.^49^ The Hospital Anxiety and Depression Scale (HADS) was used to assess depression and anxiety^50^ and the Rapid Eye Movement Sleep Behaviour Disorder Questionnaire (RBDSQ) to assess sleep.^51^ Levodopa equivalent dose scores were calculated.^52^ Participants continued their usual therapy (including levodopa) for all assessments.

Patients with Parkinson’s disease were classified as VH if they scored >1 on question 1.2 of the MDS-UPDRS Part I. We used the University of Miami Parkinson’s disease Hallucinations Questionnaire (UMPDHQ)^53^ to quantify severity of hallucinations 16 patients with Parkinson’s disease scored >1 and were classified as having Parkinson’s disease with VH, whereas 84 patients did not and were classified as non-VH. None of the Parkinson’s disease with non-VH had a history of previous hallucinations. See Table 1 for full participant demographics. The participants included in this analysis are from the same cohort as has previously been described,^54^ however they are not entirely identical due to quality control, which was completed blinded to results and exclusions based on differing analysis requirements.

**Table 1:** Participant demographics

Measure	Parkinson’s disease with no-VH (*n* = 75)	Parkinson’s disease with VH (*n* = 15)	Statistic	*P*	
Sex (F:M)	31:44	11:4	OR = 0.25	0.045	*
Age (years)	64.12 (7.78)	65.33 (8.75)	T = −0.10	0.92	ns
Years of education	16.83 (2.73)	17.77 (3.59)	U = 3311	0.27	ns
MoCA (of 30)	28.15 (2.08)	27.60 (1.76)	U = 3546	0.14	ns
UPDRS-III	21.89 (11.34)	25.40 (14.67)	U = 3331	0.38	ns
Motor dominance (left: right: both)	29:42:4	7:8:0	χ^2^ = 1.02	0.60	ns
Bionocular LogMAR visual acuity	−0.09 (0.12)	−0.04 (0.34)	U = 3325	0.34	ns
HADS depression score	3.97 (2.95)	4.73 (3.43)	U = 3334	0.39	ns
HADS anxiety score	5.77 (3.80)	7.00 (4.38)	U = 3314	0.29	ns
RBDSQ score	3.96 (2.41)	5.13 (2.53)	U = 3255	0.08	ns
Smell test (Sniffin’ Sticks)	7.92 (3.16)	6.40 (3.29)	T = 1.69	0.09	ns
Disease duration (years)	3.83 (2.38)	4.67 (2.35)	U = 3286	0.17	ns
Levodopa equivalent dose (mg)	431.89 (259.88)	421.67 (196.57)	U = 3389	0.80	ns

Means (SDs) reported. Statistical tests: OR = Fisher’s exact test odds ratio, T = two-tailed *t*-test test statistic, U = Mann–Whitney U-test test statistic, χ^2^ = chi-squared test statistic.

*P < 0.05; ns = not significant.

### Imaging protocol and pre-processing

MRI measurements were performed on a Siemens Prisma-fit 3 T MRI system using a 64-channel receive array coil (Siemens Healthcare, Erlangen, Germany). Resting-state functional MRI data consisting of gradient echo-planar imaging scans were acquired with the following parameters: TR = 70 ms (between slices); TR = 3360 ms (between volumes); TE = 30 ms; flip angle = 90°; Field-of-view = 192 × 192; voxel size = 3 × 3 × 2.5 mm; 110 volumes; scan time = 6 min. During resting-state functional MRI, participants were instructed to lie quietly with their eyes open and avoid falling asleep; this was confirmed by monitoring and post-scan debriefing. T_1_-weighted magnetization-prepared 3D rapid gradient-echo anatomical images were also acquired with the following parameters: repetition time (TR) = 2530 ms, echo time (TE) = 3.34 ms; inversion time (TI) = 1100 ms; flip angle = 7°; voxel size = 1 × 1 × 1 mm; scan time = 6 min. Imaging for all participants was performed at the same time of day (early afternoon), with participants receiving their normal medications.

The quality of resting-state functional MRI data was assessed using the MRI Quality Control tool.^55^ As resting-state functional MRI can be particularly susceptible to motion effects, stringent exclusion criteria were adopted.^56^ Specifically, participants were excluded if they met any of these criteria: (i) mean framewise displacement > 0.3 mm, (ii) any framewise displacement > 5 mm, or (iii) outliers >30% of the whole sample. This led to 10 participants being excluded ( Parkinson’s disease with VH: one patient and Parkinson’s disease with no-VH: nine patients), resulting in 90 patients being included in the functional MRI (fMRI) analysis, of whom 15 had Parkinson’s disease with VH and 75 had Parkinson’s disease with no-VH.

Resting-state data underwent standard pre-processing using SPM12. The first five volumes were discarded to allow for steady-state equilibrium. Functional data were spatially realigned, unwarped, normalized to MNI space using subject magnetization-prepared 3D rapid gradient-echo images and smoothed using a 6 mm (full width at half maximum) Gaussian kernel. Data were then denoised using independent component analysis (ICA)-based automatic removal of artefacts,^57^ which provides enhanced reproducibility of resting-state network, reduced loss of temporal degree of freedom and better conservation of signal of interest as compared with alternative noise removal strategies.^58^ ICA-based automatic removal of artefacts uses four features to classify motion components: maximum realignment parameter correlation, edge fraction, cerebrospinal fluid fraction and high frequency content, then the independent components identified as noise components are removed from the data. We used non-aggressive settings to reduce the number of nuisance regressors and avoid a possible loss of good signal or reduction in statistical power. Any residual physiological noise (and its associated uncertainty) was taken into account in the subjects’ DCMs.

### Region of interest time series extraction

**Figure 1:**
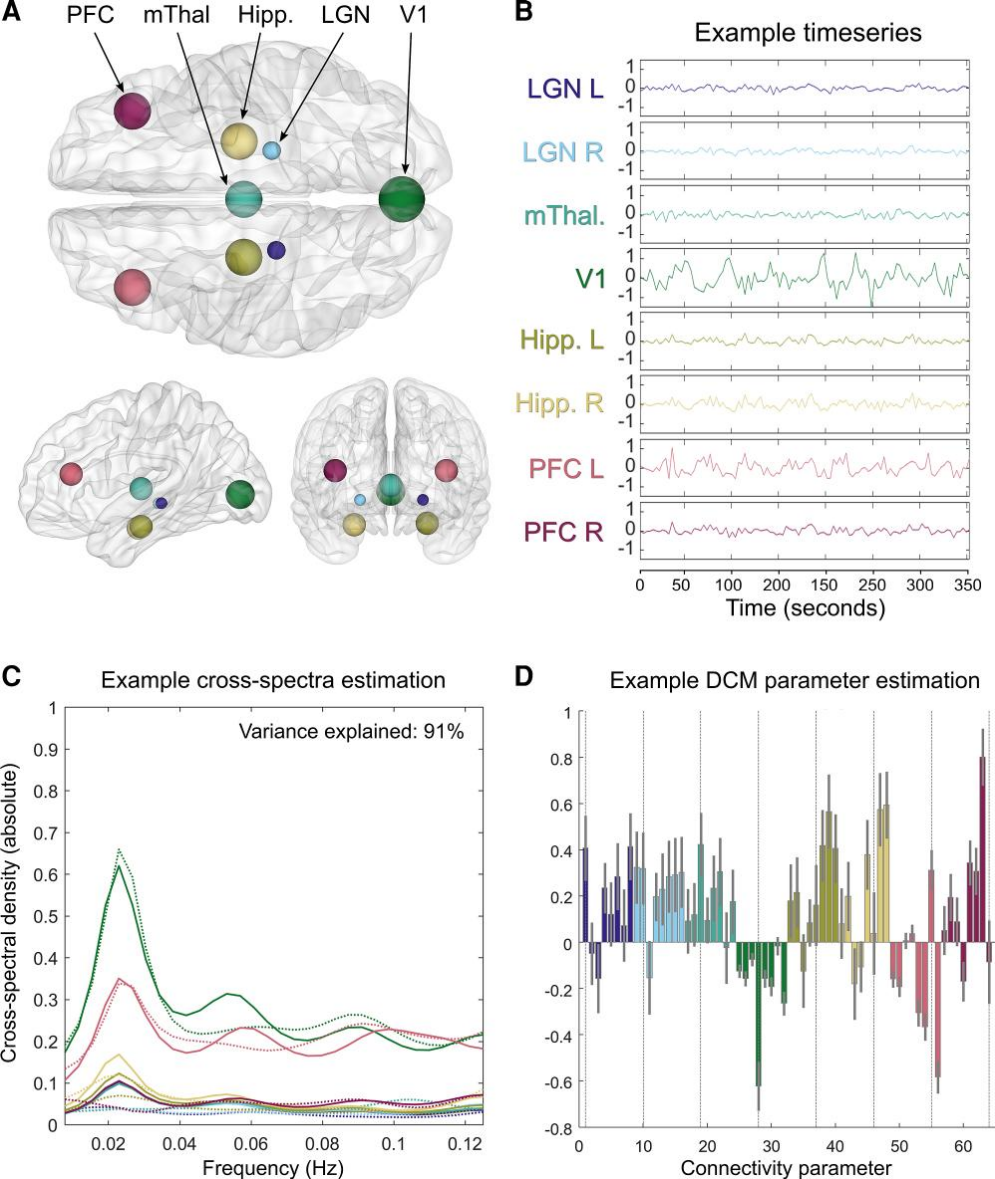
**Within-subject dynamic causal modelling analysis.** (**A**) Location of the eight nodes comprising the visual network used for all analyses (dorsal, left-sagittal and rostral views). (**B**) Time series extracted as the principal eigenvariate from each of the eight visual network nodes (example from a Parkinson’s hallucinator, data from the same patient shown for **C** and **D**). (**C**) A cross-spectral density plot, showing the frequency bands in which each of the eight regions were active. Real data are indicated by solid lines (and are second-order statistics derived from the timeseries), while data estimated by the generative model are indicated by dashed lines. The variance explained by the model for this example subject is indicated in the top right. (**D**) Estimated connectivity parameters (connection strengths) for the eight regions, including 56 extrinsic (between region) parameters, which are in hertz and eight intrinsic (within-region) parameters indicated by dotted lines, which have a unitless log scale and modulate inherently negative self-connections. Error bars are 90% credible intervals derived from the posterior variance of each parameter.

We defined a visual network comprising the following eight regions: left and right LGN, medial thalamus, primary visual cortex (V1), left and right hippocampus and left and right PFC (Fig. 1A). We included thalamic nodes in this network due to previous work showing widespread thalamic white matter tract reductions in Parkinson’s disease with VH,^20^ as well as the likely importance of thalamocortical connections in synchronizing top-down and bottom-up streams during normal visual processing.^18,19^ The LGN and V1 were selected as they are the locations of the first two post-retinal synaptic junctions within the visual system. Changes in V1 activation have been reported in Parkinson’s disease with VH on multiple occasions^28-30^ and lesion network mapping works has highlighted the LGN as the centre of overlapping networks associated with Parkinson’s disease with VH.^21^ We selected the hippocampus due to reports of a high burden of Lewy-related pathology in this region^13,59^ and previous work describing changes in functional connectivity of the hippocampus in Parkinson’s disease with VH.^26^ We selected a dorsolateral node in PFC due to this region’s role in control of visual attention^60^ and its association with (top-down) causal prediction error signal.^16,17^ Finally, the medial thalamus was selected due to its role in modulating PFC activity^61^ and because our work has shown that the medial thalamus specifically shows white matter reductions in early-stage Parkinson’s disease with VH.^20^

Using SPM12, the resting-state data for each subject was modelled using a general linear model containing a discrete cosine basis set with frequencies ranging from 0.0078 to 0.1 Hz. Data were high pass filtered to remove any slow frequency drifts (<0.0078 Hz) and an F-contrast was specified across the discrete cosine basis functions, producing a statistical parametric map (SPM) that identified regions exhibiting Blood Oxygen Level-Dependent (BOLD) fluctuations within the frequency band specified.

Locations for the LGN (MNI coordinates left, right: −22 −29 −4, 21 −27 −4), medial thalamus (MNI coordinates midline: 0 −15 6) and hippocampus (MNI coordinates left, right: −25 −15 −20, 25 −13 −21) were determined by structurally segmenting the thalamus and hippocampus in MNI space using Bayesian algorithms^62,63^ and calculating the centre of gravity for the relevant regional masks with FSL 6.0 software. Coordinates for V1 (MNI coordinates midline: 0 −83 2) were taken from a previous DCM study^64^ and those for the PFC (MNI coordinates left, right: −38 33 16, 38 33 16) from a large-scale cortical functional connectivity study.^65^ For each subject, time series were acquired by computing the principal Eigen variate of signals from spheres centred on voxels at the above coordinates (Fig. 1B). Sphere radii were 10 mm for V1, 8 mm for medial thalamus, hippocampus, PFC and 4 mm for LGN. All region of interests (ROIs) were additionally masked with the brain masks generated by fMRI-prep for each subject.

### Within-subject analysis

Effective connectivity was estimated using spectral DCM implemented in SPM12 (www.fil.ion.ucl.ac.uk/spm). For each participant, a fully connected first level (within-subject) generative model of their fMRI time series was specified, considering all possible connections among the eight ROIs. These models were then inverted using the variational Laplace scheme, which finds model parameters that optimize the trade-off between explaining the data and minimizing model complexity.^66^ This is quantified by the log model evidence, which in DCM is approximated by the free energy, which serves as the basis for comparing models. Spectral DCM significantly simplifies the model inversion by replacing the original time series with their second-order cross-spectra, meaning time-invariant parameters, rather than time-variant parameters, can be estimated.^41,44^ For inversion, we used the default prior probability densities provided by SPM for the neural, haemodynamic and state noise parameters. Further technical detail on spectral DCM can be found in the Supplementary Methods.

### Group-level analysis

#### Parametric empirical Bayes

Once optimal within-subject parameters, including connection strengths, were estimated, these were taken to the group level. Here, individual differences were modelled as hypothesized group-level effects using parametric empirical Bayes (PEB).^67^ In this hierarchical framework, constraints on the posterior density of model parameters at a given level are enforced by the level above. This analysis differs from hierarchical linear regression modelling in a frequentist setting, in that the full posterior probability densities over the parameters (their expected values and covariance) are conveyed between levels and are used to inform the results. We modelled the following in the group-level design matrix: group mean, presence of VH, age and sex. All results were thus adjusted for age and sex within the PEB model. Default prior probability densities provided by SPM12 were used for the group-level neural parameters. Further technical detail on PEB can be found in the Supplementary Methods.

#### Hypothesis-based analysis

**Figure 2:**
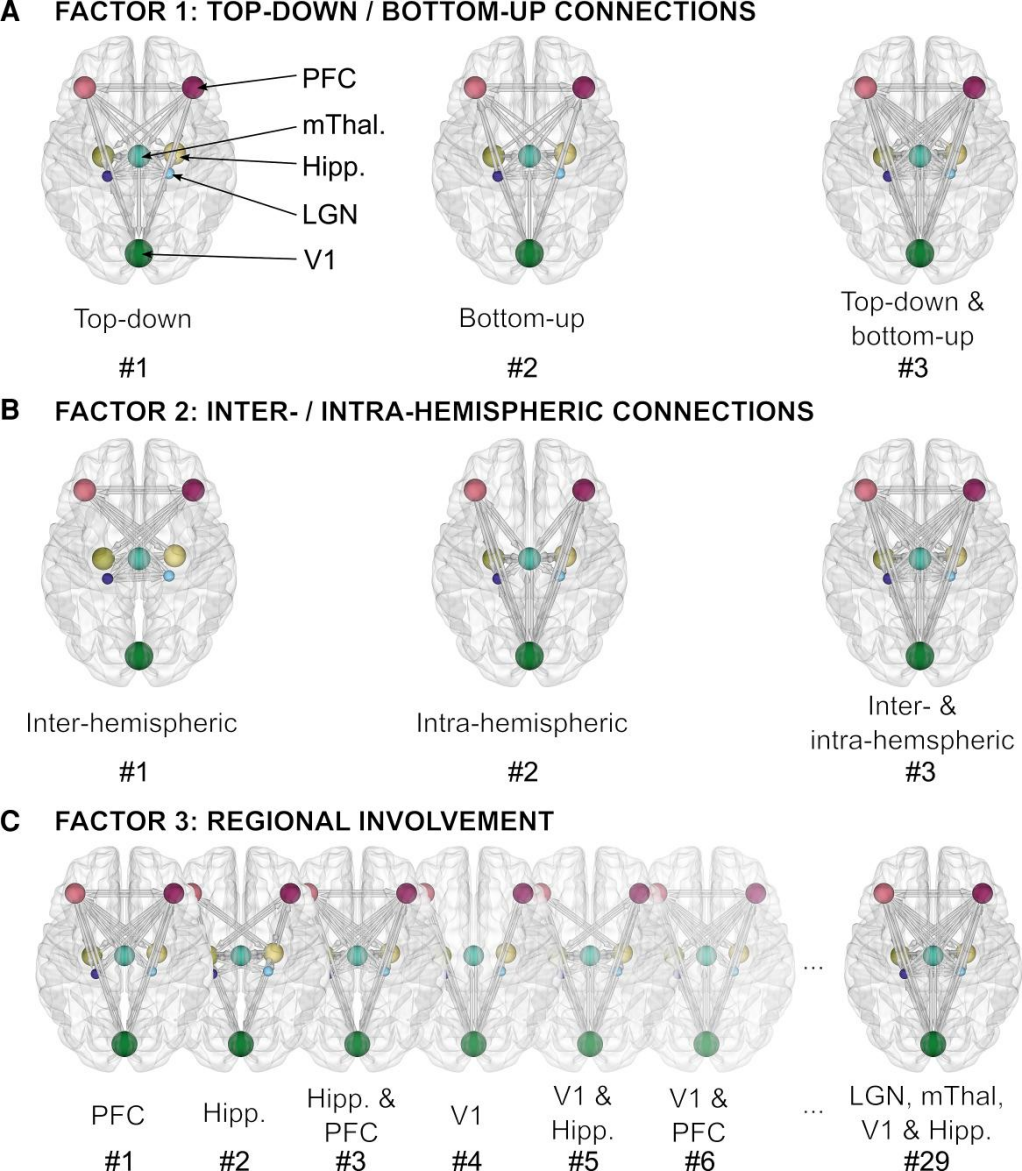
**Construction of factorial model space to test hypotheses about visual network hierarchical processing and architecture in Parkinson’s disease with VHs.** Possible explanations for the data in the form of reduced models (i.e. some connections ‘switched off’/restricted to their prior expectation of zero) were constructed based on their correspondence to three experimental factors for both the commonalities and differences between subjects. (**A**) Factor 1 examines top-down versus bottom-up connectivity and has three families within it: (i) *Top-down:* bottom-up connections switched off, (ii) *Bottom-up:* top-down connections switched off and (iii) *Top-down and bottom-up:* no connections switched off/fully connected. (**B**) Factor 2 examines interhemispheric versus intrahemispheric connectivity and has three families within it: (i) *Interhemispheric:* intra-hemispheric connections switched off, (ii) *Intrahemispheric* interhemispheric connections switched off and (iii) *Interhemispheric and Intrahemispheric:* no connections switched off/fully connected. (**C**) Factor 3 examines regional involvement and has 29 families within it, which were derived from all possible subsets of five bilateral regions (left and right LGN, medial thalamus, V1, left and right hippocampus, left and right PFC). For example in #1, all extrinsic connections are switched off except those to and from the left and right PFC and in #29, all extrinsic connections are switched on except those specific to the left and right PFC. All possible familial combinations across factors gave 3 × 3 × 29 = 261 possible models. After the removal of duplicate models and the addition of a null model with no connections switched on (including intrinsic ones), the final factorial model space contained 179 models each for the commonalities and differences between subjects. Therefore, 179^2^ = 32 041 possible hypotheses (PEB models) for the data were analysed.

We investigated the relative contributions of top-down and bottom-up connections, interhemispheric and intrahemispheric connections, as well as the involvement of different regions to the differences between subjects due to the presence of VH. To do so, we designed a set of pre-defined hypotheses in the form of reduced GLMs with certain combinations of connection parameters switched off (i.e. prior expectation set to zero) to frame each hypothesis. These candidate models were formed by the combination of three experimental factors (Fig. 2). The first factor contained three families as follows: (i) bottom-up connections off, (ii) top-down connections off and (ii) neither top-down nor bottom-up connections off (equivalent to full connectivity). The top-down and bottom-up connections were defined based on the following hierarchy (from lower- to higher-level): LGN < V1 < hippocampus < medial thalamus < PFC. The second factor contained three families: (i) intrahemispheric connections off, (ii) interhemispheric connections off and (iii) neither interhemispheric nor intrahemispheric connections off (equivalent to full connectivity). The third factor contained 29 families resulting from the 32 possible subsets of five (2^5^ = 32) bilateral regions (LGN, medial thalamus, V1, hippocampus and PFC), with the null and duplicate models removed. Combination across these factors gave 3 × 3 × 29 = 261 possible models and 179 after removal of duplicate models and addition of a null model with no connections switched on. This resulted in a final factorial model space of 179^2^ = 32 041 reduced GLMs, with 179 possible models to explain the commonalities across participants and 179 possible models to explain the differences between participants due to the presence of VH. Using Bayesian model comparison, the evidence for each individual model, as well as the pooled evidence across the families within each factor, was assessed. To obtain numerical estimates for each connection parameter, Bayesian model averaging^68^ was used to average parameter values across all models (weighted by the models’ posterior probabilities). Only parameters with >95% posterior probability of being present versus absent are reported.

#### Automatic parameter search

To test the validity of the network structure suggested by our hypothesis-based analysis, we additionally used a data-driven approach in the form of an automatic (greedy) search over connection parameters. This algorithm automatically switched off connection parameters from the full model until there was no increase in free energy.^69,70^ The parameter values from the 256 models from the final iteration of the algorithm were averaged and weighted by their model evidence (Bayesian model averaging). Only parameters with >95% posterior probability of being present versus absent are reported. BrainNet Viewer was used to visualize data on cortical surfaces (https://www.nitrc.org/projects/bnv/).^71^

#### Leave-one-out cross-validation

We next investigated whether subjects’ group membership (Parkinson’s disease with VH or Parkinson’s disease with no-VH) could be predicted from their connectivity parameters, i.e. whether changes in effective connectivity between subjects were predictive of the presence of VH. We applied leave-one-out cross-validation across all subjects, fitting a PEB model to all but one participant each time and predicting the group effect for the left-out subject based on their individual connectivity parameters. Across subjects, we used the five connections that showed the greatest difference between Parkinson’s disease with VH and Parkinson’s disease with no-VH at group level as identified by the Bayesian model average over our factorial model space. These were (in descending order): left LGN to V1, left PFC to V1, right PFC to V1, left LGN to left PFC and left PFC to medial thalamus. To ensure that our findings were not dependent on a specific number of connections, we also replicated our results using the top 10 connection strengths and the single largest connection strength.

#### Canonical variate analysis

Finally, we investigated whether, within the Parkinson’s disease with VH group, hallucination severity (as measured by the UMPDHQ hallucinations severity scale) was associated with the pattern of effective connectivity seen. To do so, we applied canonical variate analysis (CVA), to model the effect of the same five connection strengths described above against the UMPDHQ score for the 15 Parkinson’s disease subjects with VH. CVA allowed dimension reduction of the five parameters into two canonical variates of connectivity. We tested the correlation between the first canonical variate of connectivity and the first canonical variate of hallucination severity, adjusted for age and sex. We replicated this analysis using the top 10 and the single largest connection strength.

### Statistical analyses

Differences in demographics and clinical characteristics between groups were examined with independent sample *t*-tests for normally distributed continuous variables, Kruskal–Wallis tests for non-normally distributed ones, Fisher’s exact tests for categorical variables with two categories and chi-squared tests for categorical variables with three categories. The Lilliefors test was used to assess normality. Subject-level DCMs were inverted using variational Laplace.^66^ PEB^67^ was used to estimate group-level parameters from subject-level DCMs. Bayesian model comparison^72^ was used to compare the evidence for different models and Bayesian model averaging was used to generate weighted average connection strengths across models.^68^ Leave-one-out cross validation was used to predict group membership and CVA was used to test the association between connectivity and hallucination severity. The point biserial correlation was used to test associations between dichotomous and continuous variables, while the Pearson correlation coefficient was used to test associations between two continuous variables.

## Results

### Participants

Of the 90 Parkinson’s disease patients in the current study, 15 were identified as having VH (Parkinson’s disease with VH), while 75 had no-VH (Parkinson’s disease with no-VH). The Parkinson’s disease with no-VH group had a higher male to female ratio than the Parkinson’s disease with VH group (T = 3.90, *P* = 0.045), but there were no significant differences in the other demographics or clinical metrics, including no difference in measures of cognition or Levodopa equivalent dose (Table 1).

### Accuracy of dynamic causal model estimation

The variance explained by DCM model estimation when fitted to the observed spectral data was 89.6 ± 3.3% [mean ± standard deviation (SD)], with a range of 81.22–96.96%. This confirms the good fits of the estimated DCM to the empirical cross-spectra. An example, participant’s estimation across spectral densities and estimated connectivity parameters can be seen in Fig. 1C and D. Cross-spectral density plots for all 15 Parkinson’s disease subjects with VH and a summary plot across all subjects can be seen in Supplementary Fig. 1.

### Commonalities of visual network effective connectivity across participants

**Figure 3:**
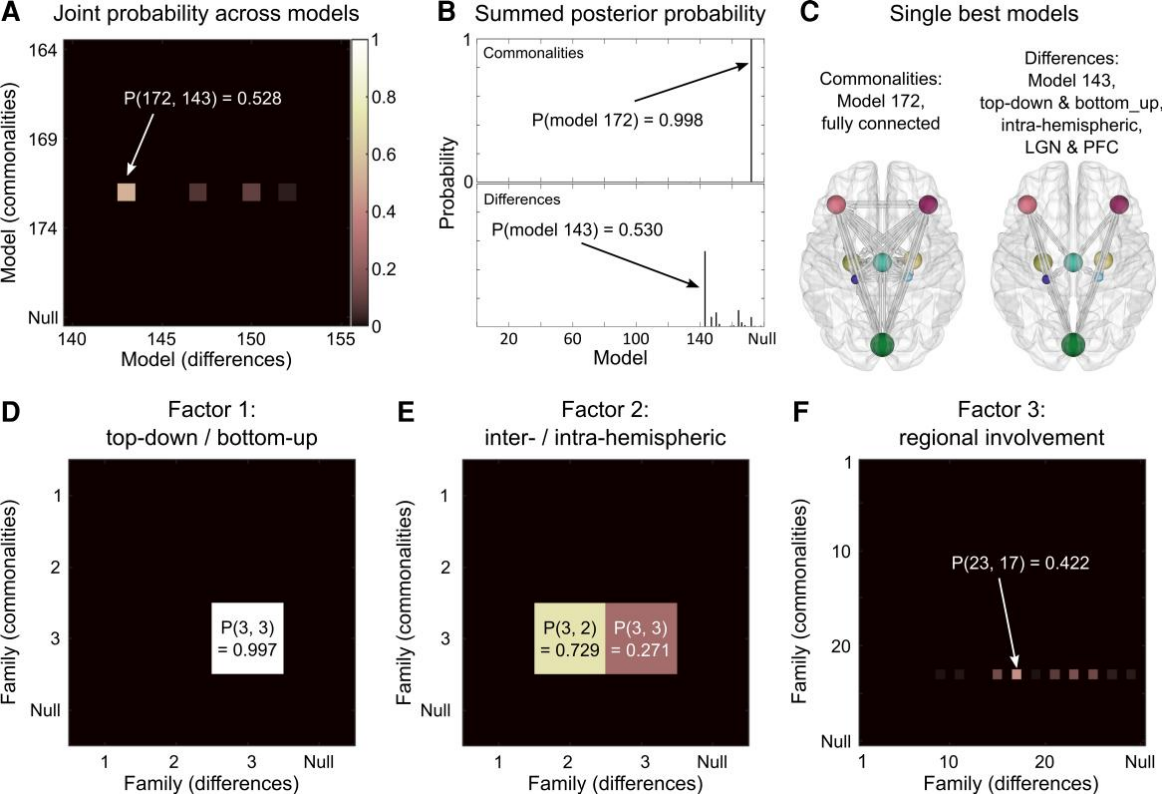
**Bayesian model comparison across factorial model space gives possible explanations for visual network hierarchical processing and architecture.** (**A**) Joint posterior probability across all models. The axes list all 179 candidate models, including the null model, in terms of the commonalities across subjects and differences due to the presence of VHs (i.e. the hypothesis in row *i* and column *j* had parameters relating to commonalities set according to Model *i* and parameters relating to the presence of hallucinations set according to Model *j*). The best model was number 172 (fully connected) for the commonalities and number 143 (top-down and bottom-up with intrahemispheric LGN and PFC) for the differences, with a 53% posterior probability. (**B**) Summed posterior probability. The same result shown in **A**, summed over the columns to give the posterior probability for the commonalities across subjects and summed over the rows and to give the posterior probability for the differences due to hallucinations. (**C**) Spatial projections for the two single best models described in **A** and **B**. (**D**–**F**) Family analyses for each factor: **D**: top-down/bottom-up; **E**: interhemispheric/intrahemispheric; **F**: regional involvement. Separate family analyses with the same 32 041 hypothesis (reduced PEB models) from **A** grouped into different families. In each case, the element in row *m* and column *n* represents the pooled probability across models in which the commonalities parameters were set according to family *m* and the parameters relating to the presence of hallucinations were set according to family *n*. Factors and families are as described in Fig. 2 and the probability associated with the null model is also plotted in each case.

The visual network architecture common to all participants with Parkinson’s disease was defined by the commonalities in connection strengths, or group mean, identified in our hypothesis-based analysis. Across all reduced GLMs, the highest associated posterior probability was 53% (Fig. 3A). This GLM had its commonalities quantified by the effective connectivity parameters of model 172 (Fig. 3C, left panel), which was the fully connected model (all connections switched on). Summing across all GLMs, which had their commonalities parameters deployed according to this model, the posterior probability reached 99.8% (Fig. 3B, *upper panel*). This strong evidence indicates that the commonalities across subjects were best explained by the fully connected model. Thus, no connections could be excluded without reducing the quality of the model. Accordingly, family-based analyses revealed that the families reflecting full connectivity within a factor were important in explaining the commonalities across subjects. These families are: both top-down and bottom-up connections, (Family 3 in Factor 1, Fig. 3D), both interhemispheric and intrahemispheric connections (Family 3 in Factor 2, Fig. 3E) and connections to and from all regions (Family 23 in Factor 3, Fig. 3F).

**Figure 4:**
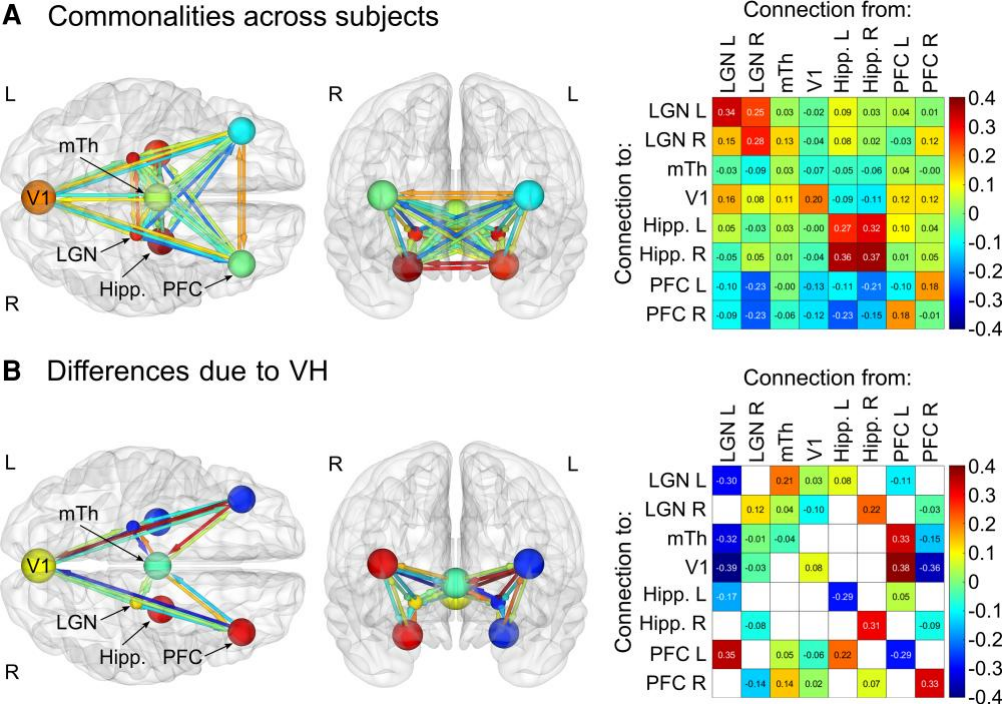
**Bayesian model averaging across factorial model space to give the magnitude of connection strengths across subjects and differences associated with VHs.** The Bayesian model average of parameter values across all 179 models accounts for the commonalities between subjects and the differences between subjects due to the presence of VHs. All parameters are thresholded at posterior probability >95% of being present versus absent. (**A**) Commonalities across all patients (with and without hallucinations, equivalent to the mean across subjects). Here, off-diagonal positive numbers (represented by orange arrows on the left) reflect excitatory connectivity and off-diagonal negative numbers (represented by blue arrows on the left) reflect inhibitory connectivity. Diagonal connectivity/self-connectivity are inhibitory by definition and are measured on a log scale, hence, leading diagonal positive numbers (represented by orange spheres on the left) reflect more self-inhibition and leading diagonal negative numbers (represented by blue spheres on the left) reflect disinhibition. (**B**) Differences between VH and non-VH. Here, off-diagonal positive numbers (represented by orange arrows on the left) reflect increased connectivity in Parkinson’s disease with VH versus Parkinson’s disease with no-VH, whereas off-diagonal negative numbers (represented by blue arrows on the left) reflect decreased connectivity. Leading diagonal positive numbers (represented by orange spheres on the left) reflect increased self-inhibition in Parkinson’s disease with VH versus Parkinson’s disease with no-VH and leading diagonal negative numbers (represented by blue spheres on the left) reflect increased disinhibition.

Bayesian parameter averaging of commonalities between subjects revealed that this fully connected model was characterized by strongly positive interhemispheric effective connectivity and strongly positive intrinsic (self) connectivity in all regions except the PFC and medial thalamus. Note that effective connectivity with a positive sign represents excitatory influences and a negative sign represents inhibitory influences except for (log-scaled) self-connections, which are always inhibitory by definition. Hence, positive self-connections represent more inhibition and negative self-connections represent disinhibition. Other key features included generally negative afferent PFC connectivity and positive connectivity to V1 from LGN and PFC (Fig. 4A). This architecture was confirmed by our data-driven automatic search over parameters, which identified a highly connected network with very similar connection strengths (Supplementary Fig. 2).

### Effect of visual hallucinations on visual network architecture

The differences in visual network architecture in Parkinson’s disease with VH compared with Parkinson’s disease with no-VH were best explained by GLMs with parameters deployed according to Model 143 (Fig. 3A), with an associated summed posterior probability of 53% (Fig. 3B, *bottom*). This model was defined by the presence of both top-down and bottom-up intrahemispheric connections to and from LGN to PFC (Fig. 3C, right panel). Accordingly, family-based analysis revealed that both top-down and bottom-up connections (Family 3 in Factor 1, Fig. 3D), intrahemispheric connections (Family 2 in Factor 2, Fig. 3E) and connections to and from LGN and PFC (Family 17 in Factor 3, Fig. 3F) were the best explanation for differences between subjects due to the presence of VH.

While model 143 had the strongest posterior probability across all 179 models, its overall probability was only 53%. There was therefore not a single best explanation for the differences in connectivity due to VH (i.e. a model with 95% probability or more^73,74^). This *dilution of the evidence* effect is expected given the large number of models. Therefore, to summarize the estimated parameters across the 32 041 models, while taking into account that different models had different levels of evidence, we performed Bayesian model averaging. We then thresholded the averaged parameters at >95% posterior probability, corresponding to strong evidence,^73,74^ for effects being present versus absent.

A Bayesian model averaging across all models in the factorial model space revealed several key differences in effective connectivity in Parkinson’s disease with VH compared with Parkinson’s disease with no-VH. These included reduced effective connectivity from left LGN to V1 (0.39 Hz) and medial thalamus (0.32 Hz), increased effective connectivity from left LGN to left PFC (0.35 Hz), increased effective connectivity from left PFC to medial thalamus (0.33 Hz) and V1 (0.38 Hz) and decreased effective connectivity from right PFC to V1 (0.36 Hz). Additionally, in Parkinson’s disease with VH, we observed increased self-inhibition in the right PFC and right hippocampus and disinhibition of the left PFC, left hippocampus and left LGN. The full pattern of connectivity differences can be seen in Fig. 4B. The differences between subjects due to the presence of VH identified after an automatic search over parameters were in concordance with those described above (Supplementary Fig. 2).

This indicates that both increased top-down and reduced bottom-up effective connectivity are important in explaining the differences between Parkinson’s disease with VH and Parkinson’s disease with no-VH groups, particularly relating to the LGN and PFC, with a lateralized effect observed from the PFC.

#### Predicting group membership and hallucination severity

**Figure 5:**
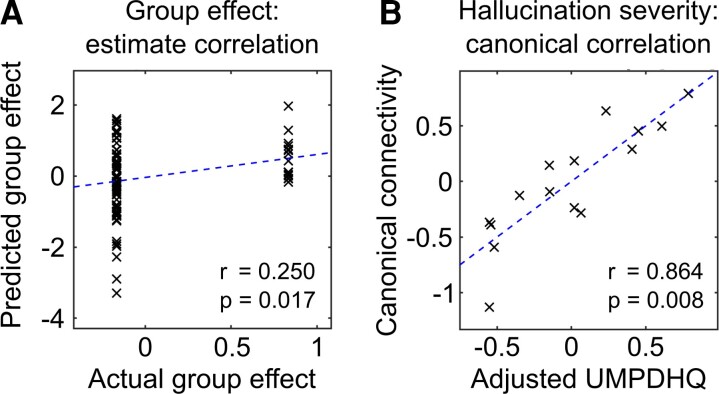
**Visual network effective connectivity is associated with the presence and severity of VHs in Parkinson’s disease.** (**A**) Results from leave-one-out cross validation across all 90 subjects. The five largest parameters in terms of overall group difference were used to predict the left-out subject’s group membership. The correlation between the predicted (demeaned) group effect and the actual (demeaned) group effect, *r* is the point biserial correlation coefficient. (**B**) Relationship between differences in effective connectivity and hallucination severity. Results of CVA for the 15 Parkinson’s disease with VH. For each participant, the primary canonical variate derived from their individual values for the five largest parameters in terms of overall group difference between Parkinson’s disease with VH and Parkinson’s disease with no-VH is plotted against the primary canonical variate derived from their UMPDHQ score adjusted for age and sex, *r* is the Pearson’s correlation coefficient.

We used leave-one-out cross validation to test whether a participant’s group membership (i.e. hallucination status) could be predicted from their individual connection strengths for the top five largest group differences, as previously described in the Materials and methods section. We found that 68 out of 90 subjects had their true group membership value within the estimated 90% credible interval, with 13 of 15 Parkinson’s disease with VH and 55 of 75 Parkinson’s disease subjects with no-VH falling in this range. Predicted group membership correlated significantly with actual group membership [*r* = 0.25 (point biserial correlation coefficient), *P* = 0.017; Fig. 5A]. Additionally, this correlation remained significant when predicting group membership using the top 10 connections (*r* = 0.31, *P* = 0.003) or the single largest connection (*r* = 0.23, *P* = 0.025).

We next investigated whether hallucination severity within the Parkinson’s disease with VH group was associated with the first canonical variate derived from the five parameters described above. We found that this correlated significantly with the canonical variate derived from the adjusted UMPDHQ score [*r* = 0.864 (Pearson’s correlation coefficient), *P* = 0.008; Fig. 5B], indicating that the pattern of connectivity within the visual network in the Parkinson’s disease with VH group was predictive of hallucination severity. This association remained significant when using the top 10 connection strengths (*r* = 0.854, *P* = 0.018) and approached significance when using the single largest connection (*r* = 0.643, *P* = 0.053).

## Discussion

In the current study, we utilized spectral DCM for resting-state fMRI data to investigate visual network effectiveness and connectivity in Parkinson’s disease patients with and without VH. We examined how effective connectivity differed between patients with and without VH, finding that a model comprising both reduced bottom-up and increased top-down effective connectivity best explained differences between patient groups, with differences in effective connectivity relating to the severity of hallucinations. To our knowledge, this is the first study demonstrating changes in causal influences between brain regions in Parkinson’s disease VH.

The ability of DCM to non-invasively infer the directionality of influences between brain regions in living patients^75^ ideally places it to investigate hypotheses relating to the hierarchical processing account of VH, where reduced bottom-up sensory processing is proposed to occur alongside overweighting of top-down perceptual priors.^14,15^ Previous studies, while making valuable insights,^27,76^ have tended to look at functional connectivity changes, which are inherently undirected in nature^75^ and so cannot directly test this hypothesis. We designed a factorial model space to specifically probe the relative contribution of top-down and bottom-up connections within the visual network in Parkinson’s disease with VH versus Parkinson’s disease with no-VH and additionally tested interhemispheric versus intrahemispheric connectivity alongside regional involvement to estimate the architecture of any changes. Finally, we assessed whether the pattern of visual network changes observed was associated with a clinically relevant measure: the severity of hallucinations.

Our Bayesian model comparison across this model space revealed that the differences between subjects due to the presence of VH were best explained by a combination of both top-down and bottom-up effective connections. In particular, these were intrahemispheric connections to and from the LGN and PFC. A Bayesian model averaging across the model space revealed both the valence and magnitude of such changes. Among the largest were decreased effective connectivity in Parkinson’s disease with VH from the left LGN to the medial thalamus and V1, as well as increased effective connectivity from the left PFC to the medial thalamus and V1. Thus, some of the largest differences we found between Parkinson’s disease with VH and Parkinson’s disease with no-VH reflected both increased top-down and reduced bottom-up connections within the visual network, with regions at either end of the visual hierarchy (LGN and PFC) emerging as particularly important.

The single largest difference we found between Parkinson’s disease with VH and Parkinson’s disease with no-VH was decreased effective connectivity from the left LGN to V1 in Parkinson’s disease with VH. This is consistent with previous structural network mapping work, which found that coordinates of atrophy from studies of VH in Parkinson’s disease were connected to a network centred on the LGN.^21^ Similarly, lesions in patients with VHs are connected to an LGN-centred functional network.^22^ Task-based functional MRI work in Parkinson’s disease with VH has also shown decreased activity in primary visual and occipital cortex fairly consistently in Parkinson’s disease with VH compared with Parkinson’s disease with no-VH.^28-30^ Additionally, magnetic resonance spectroscopy has revealed reduced occipital γ-Aminobutyric acid (GABA)^77^ and fluorodeoxyglucose (FDG)-positron emission tomography (PET) has revealed occipital glucose hypometabolism associated with VH in Parkinson’s disease.^78,79^ Reduced activity in early visual cortex would be consistent with reduced bottom-up input from LGN, although we did not find altered efferent connectivity from V1 in Parkinson’s disease with VH. Interestingly, a recent study of effective connectivity in Parkinson’s disease without VH found that modulation of LGN by the superior colliculus in response to luminance contrast changes was inhibited in Parkinson’s disease compared with controls.^80^ The way in which such stimuli modulate the LGN and the superior colliculus may also change as a result of treatment.^81^

We observed changes in effective connectivity to the medial thalamus in Parkinson’s disease with VH, with decreased connectivity from the left LGN and increased connectivity from the left PFC. This is congruent with our recent work finding that thalamic tracts connected to the mediodorsal thalamic nucleus had reduced fibre cross section in Parkinson’s disease with VH,^20^ although these findings did not indicate the direction of information flow. Previous diffusion tensor imaging work revealed increased mean diffusivity in bilateral thalamic subregions projecting to prefrontal and parieto-occipital cortices in Lewy body dementia patients with VH.^82^ Additionally, a recent post-mortem study found that atrophy in the mediodorsal nucleus of the thalamus was significantly greater in Lewy body dementia patients with VH.^83^

The thalamus is expected to play an important role in accounts of dysfunctional hierarchical predictive processing, due to its role in synchronizing simultaneous streams of information (e.g. top-down and bottom-up) in normal visual processing.^18,19^ This is corroborated by the changes in thalamic effective connectivity we found in Parkinson’s disease with VH. Interactions between the thalamus and PFC are important in dealing with perceptual uncertainty during decision making.^84^ The two separate pathways may exist for this, with low-signal-related uncertainty (as would be expected with reduced bottom-up sensory input) resolved by dopaminergic projections from the medial thalamus that increase prefrontal output.^85^ Interestingly, changes in thalamic resting-state functional connectivity,^86^ as well as changes in thalamocortical effective connectivity^87^ may also underlie hallucinations during exposure to lysergic acid diethylamide (LSD), which is consistent with a more general role for changes in thalamic connectivity in hallucinations in other contexts.^88^

We also found increased top-down effective connectivity in Parkinson’s disease with VH from the left PFC to the medial thalamus and V1. This might help to explain previous task-based functional studies reporting increased activity in Parkinson’s disease with VH prefrontal cortices in response to simple stimuli,^28,30^ although other work has shown that PFC activity may be decreased in Parkinson’s disease with VH versus Parkinson’s disease with no-VH in response to more complex stimuli.^31,32^ Similarly, previous resting-state work has described increased functional connectivity between superior, middle and inferior frontal gyri in Parkinson’s disease with VH and occipital cortex.^39^ An increased burden of Lewy-related pathology has also been reported in frontal cortex in Parkinson’s disease with VH,^13^ as have grey matter reductions in the bilateral dorsolateral PFC.^89^

The lateralized effect of PFC efferent connectivity we found is intriguing, in that we found increased top-down connectivity from the left PFC to V1 and the medial thalamus, but decreased top-down connectivity from the right PFC to V1. Lateralized differences in prefrontal activity have also been reported in previous functional neuroimaging studies of Parkinson’s disease with VH. Both increased^78^ and decreased^79^ glucose metabolism have been observed in the left PFC. Decreased right PFC BOLD activation has also been reported in Parkinson’s disease with VH in response to faces,^31^ and just prior to the presentation of complex stimuli.^32^ Increased right PFC BOLD activation has meanwhile been reported in response to more simple stimuli.^30^ A single-case study has also reported simultaneous activation of the right medial frontal gyrus and deactivation of the left middle frontal gyrus during active VH.^90^

Asymmetry in lateral PFC influences has been shown in prediction error signalling in health, however right (rather than left) lateral PFC was implicated.^16,17^ Our observation of increased effective connectivity from left PFC and decreased from right PFC is thus inconsistent with these findings. It is possible that Parkinson’s disease disrupts the usual laterality of prediction error signals, though this needs to be tested in larger numbers of patients. We also assessed the asymmetry of motor symptoms in our cohort, but as we did not see any significant differences between the Parkinson’s disease with VH and Parkinson’s disease with no-VH group, it seems unlikely that this would drive the effect.

Although we found evidence to support the increased top-down and reduced bottom-up connectivity account of VH in Parkinson’s disease, differences in effective connectivity between other regions are also implicated. For example, we also observed increased bottom-up connectivity from the left LGN and decreased top-down connectivity from the right PFC in Parkinson’s disease with VH and this is further complicated by changes in regional self-connections. We found disinhibition of the left LGN (reduced intrinsic self-inhibition), which would be expected to amplify both the reduced bottom-up connectivity to the medial thalamus and V1 and the increased bottom-up connectivity to the left PFC. We further found disinhibition of the left PFC, which would amplify its increased top-down connections to the medial thalamus and V1 and we found increased self-inhibition of the right PFC, which would dampen its reduced top-down connection to V1. Of note, the combined differences in connectivity are related to clinical measures of hallucination severity (both top 5 and top 10 connections), so a more complex shift in causal influences may be relevant. It is therefore important to consider the full set of changes in effective connectivity as well as both extrinsic and intrinsic connections when interpreting our results.

### Limitations

One of the main limitations of the current paper is the relatively small number of cases of Parkinson’s disease with VH included. While similar sample sizes have been used in previous functional analyses of VH in Parkinson’s disease,^34,91^ it will be important to examine effective connectivity in larger cohorts.

Patients in the current study continued their normal medication, including levodopa, during both clinical assessments and neuroimaging. This was done to avoid the potential effects of distress and anxiety caused by omitting levodopa doses, which would affect cognitive function. Although we are not able to directly assess the effect of neurotransmitters on the current results, we note there was no significant difference in levodopa equivalent dose between VH and non-VH.

Additionally, while predicted group membership correlated significantly with actual group membership in our leave-one-out analysis, we were not able to predict group membership on a per-subject basis with a high posterior probability. This could be explained by heterogeneity within the Parkinson’s disease with no-VH group, whereby those who will go on to develop VH show a similar pattern to those who already have VH. However, longitudinal data would be needed to test this. That said, we did find that the pattern of connectivity was associated with hallucination severity within the Parkinson’s disease with VH group.

## Conclusions

Our spectral DCM analysis showed that VH in Parkinson’s disease is associated with both reduced bottom-up and increased top-down effective connectivity within the visual network. This particularly related to intrahemispheric connectivity to and from the LGN and PFC. The pattern of effective connectivity within the Parkinson’s disease with VH group was significantly associated with the severity of their hallucinations. This study provides further evidence for the aberrant hierarchical predictive processing account of hallucinations in Parkinson’s disease and models resting-state effective connectivity for the first time in this context.

## Supplementary Material

fcac329_Supplementary_Data

## Data Availability

Anonymized, group-level summary DCM data and code for performing group-level analyses are available on github (https://github.com/gecthomas/Spectral_DCM_in_PD_VH).

## Abbreviations

CVA =: canonical variates analysis
DCM =: dynamic causal modelling
LGN =: lateral geniculate nucleus
Parkinson’s disease with no-VH =: Parkinson’s disease without visual hallucinations
PD-VH =: Parkinson’s disease with visual hallucinations
PEB =: parametric empirical Bayes
PFC =: prefrontal cortex
V1 =: primary visual cortex
VH =: visual hallucination
