# Supplementary material for: Changes in both top-down and bottom-up effective connectivity drive visual hallucinations in Parkinson’s disease
Source: Brain Commun. 2022 Dec 14;5(1):fcac329. doi: 10.1093/braincomms/fcac329 (PMC9798302; doi:10.1093/braincomms/fcac329)
Supplement: fcac329_Supplementary_Data [file fcac329_supplementary_data.pdf]

Supplementary materials

Methods:

Spectral Dynamic Causal Modelling

Dynamic causal modelling (DCM) is a Bayesian framework that infers the directed (causal) connectivity among the neuronal systems – referred to as effective connectivity. A new DCM for resting state fMRI was recently proposed – based upon a deterministic model that generates predicted cross spectra – referred to as spectral DCM. In order to model resting state activity – in the absence of external stimuli – a stochastic component, i.e. neural fluctuations, is added to the classical DCM based on ordinary differential equations. Mathematically, we can express the formulation of the stochastic generative model using a set of two equations. First is the neuronal state equation, namely

$$\dot{x}(t) = f(x(t), u(t), \theta) + v(t), \qquad \text{(S1)}$$

and second is the observation equation, which is a static nonlinear mapping from the hidden physiological states in (1) to the observed BOLD activity and is written as:

$$y(t) = h(x(t), \varphi) + e(t), \qquad \text{(S2)}$$

where $\dot{x}(\mathrm{t})$ is the rate of change of the neuronal states $x(t)$, θ are unknown parameters (i.e. the effective connectivity) and $v(t)$ (resp. $e(t)$) is the stochastic process – called the state noise (resp. the measurement or observation noise) – modelling the random neuronal fluctuations that drive the resting state activity. In the observation equations, φ are the unknown parameters of the (haemodynamic) observation function and $u(t)$ represents any exogenous (or experimental) inputs that drive the hidden states – that are usually absent in resting state designs (Friston *et al.*, 2014). Spectral DCM furnishes a constrained inversion of the stochastic model by parameterising the neuronal fluctuations $v(t)$. Spectral DCM simplifies the generative model by replacing the original timeseries with their second-order statistics (i.e., cross

spectra). This means, instead of estimating time varying hidden states, we are estimating their covariance which is time invariant. Then we simply need to estimate the covariance of the random fluctuations; where a scale free (power law) form for the state noise (resp. observation noise) is used – motivated from previous work on neuronal activity (Beggs and Plenz, 2003; Shin and Kim, 2006; Stam and de Bruin, 2004) – as follows:

$$g_v(\omega, \theta) = \alpha_v \omega^{-\beta_v}$$

$$g_e(\omega, \theta) = \alpha_e \omega^{-\beta_e} \qquad \text{(S3)}$$

Here, $\{\alpha, \beta\} \subset \theta$ are the parameters controlling the amplitudes and exponents of the spectral density of the neural fluctuations. The parameterisation of endogenous fluctuations means that the states are no longer probabilistic; hence the inversion scheme is significantly simpler, requiring estimation of only the parameters (and hyperparameters) of the model.

We used standard Bayesian model inversion to infer the parameters of the model in (1), (2) and (3), from the observed signal $y(t)$. The description of the Bayesian model inversion procedures based on variational Laplace can be found elsewhere for the interested readers (Friston *et al.*, 2007; Friston *et al.*, 2003; Razi and Friston, 2016).

Parametric Empirical Bayes

Empirical Bayes refers to the Bayesian inversion or fitting of hierarchical models. In hierarchical models, constraints on the posterior density over model parameters at any given level are provided by the level above. These constraints are called empirical priors because they are informed by empirical data. We recently introduced a second-level or between-subjects model over parameters, which represents how individual (within-subject) connections derive from the subjects' group membership (Friston et al., 2016) – based on parametric empirical Bayes (PEB). This approach calls on Bayesian Model Reduction (BMR) to finesse the inversion of multiple models of a single dataset

or a single (hierarchical) model of multiple datasets. BMR allows one to compute posterior densities over model parameters, under new prior densities, without explicitly inverting the model again. For example, one can invert a DCM for each subject in a group and then evaluate the posterior density over group effects, using the posterior densities over parameters from the single subject inversion. This may improve subject-specific parameter estimates, by using group-level estimates to rescue individual DCM from local optima. Mathematically, for DCM studies with *N* subjects and *M* parameters per DCM, we have a hierarchical model, where the responses of the *i*-th subject and the distribution of the parameters over subjects can be modeled as:

$$y_i = \Gamma_i^{(1)}(\theta^{(1)}) + \varepsilon_i^{(1)} \qquad \text{(S4)}$$

$$\theta^{(1)} = \Gamma^{(2)}\left(\theta^{(2)}\right) + \varepsilon^{(2)}$$

$$\theta^{(2)} = \eta + \varepsilon^{(3)}$$

where, y_i is the BOLD time series from *i-th* subject *and* $\Gamma_i^{(1)}$ is a nonlinear mapping from the parameters of a model to the predicted response y for e.g. as shown in Eq. S1 above. $\varepsilon_i^{(1)}$ is independent and identically distributed (i.i.d.) observation noise (equivalent to $e(t)$ in Eq. S2). In this hierarchical form, *empirical priors* encoding second (between-subject) level effects place constraints on subject-specific parameters. The second level would be a linear model where the random effects are parameterised in terms of their precision:

$$\Gamma^{(2)}\left(\theta^{(2)}\right) = (X \otimes W)\beta$$

where, $\beta \subset \theta$ are group means or effects encoded by a design matrix with between X and within-subject W parts. The between-subject part encodes differences among subjects or covariates such as age, while the within-subject part specifies mixtures of parameters that show random effects. We assume that the first column of the design matrix is a constant term, modelling group means and subsequent columns encode group differences or covariates such as age.

References:

Beggs JM & Plenz D. 2003. Neuronal avalanches in neocortical circuits. J Neurosci 23:11167-11177.

Friston K, Mattout J, Trujillo-Barreto N, Ashburner J, & Penny W. 2007. Variational free energy and the Laplace approximation. Neuroimage 34:220-234.

Friston KJ, Harrison L, & Penny W. 2003. Dynamic causal modelling. Neuroimage 19:1273-1302.

Friston KJ, Kahan J, Biswal B, & Razi A. 2014. A DCM for resting state fMRI. Neuroimage 94:396-407.

Friston KJ, Litvak V, Oswal A, Razi A, Stephan KE, van Wijk BC*, et al.* 2016. Bayesian model reduction and empirical Bayes for group (DCM) studies. Neuroimage 128:413-431.

Razi A & Friston K. 2016. The Connected Brain: Causality, models, and intrinsic dynamics. IEEE Signal Processing Magazine 33:14-35.

Shin CW & Kim S. 2006. Self-organized criticality and scale-free properties in emergent functional neural networks. Phys Rev E Stat Nonlin Soft Matter Phys 74:045101.

Stam CJ & de Bruin EA. 2004. Scale-free dynamics of global functional connectivity in the human brain. Hum Brain Mapp 22:97-109.

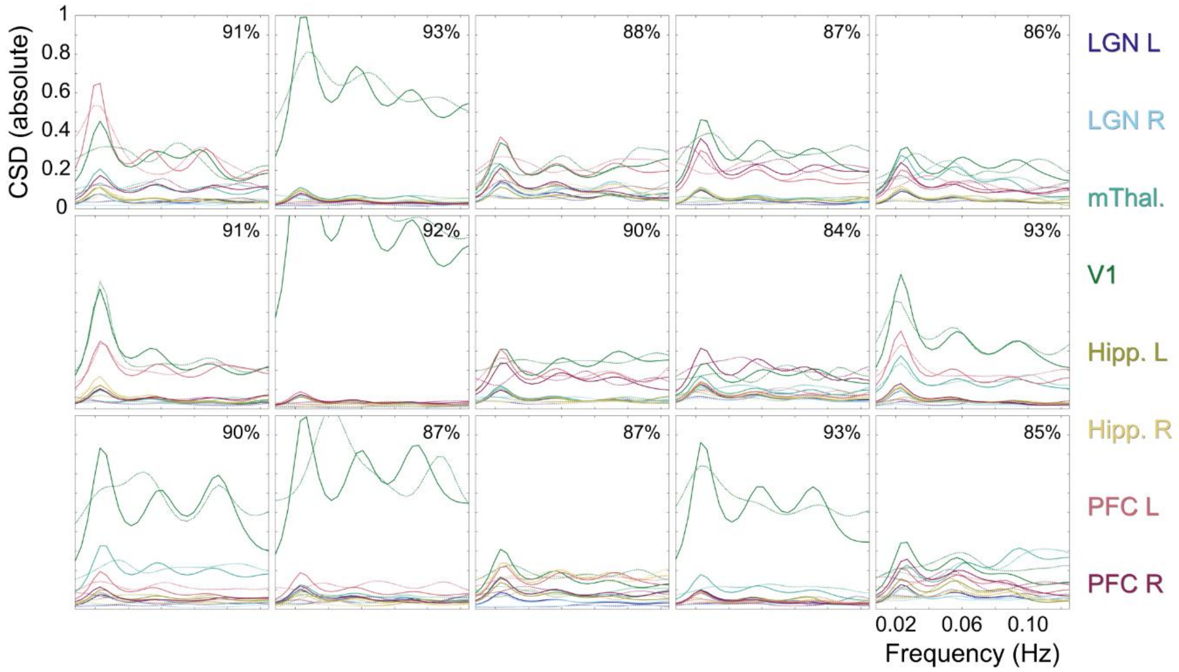

Supplementary figure 1 - Cross-spectral density (CSD) plots for the 15 PD-VH subjects showing the frequency bands in which each of the eight regions were active. Real data are indicated by solid lines (and are second order statistics derived from the timeseries), while data estimated by the generative model are indicated by dashed lines. The variance explained by each subject's dynamic causal model is indicated in the top right. LGN = lateral geniculate nucleus; mThal = medial thalamus; Hipp. = hippocampus; PFC = prefrontal cortex.

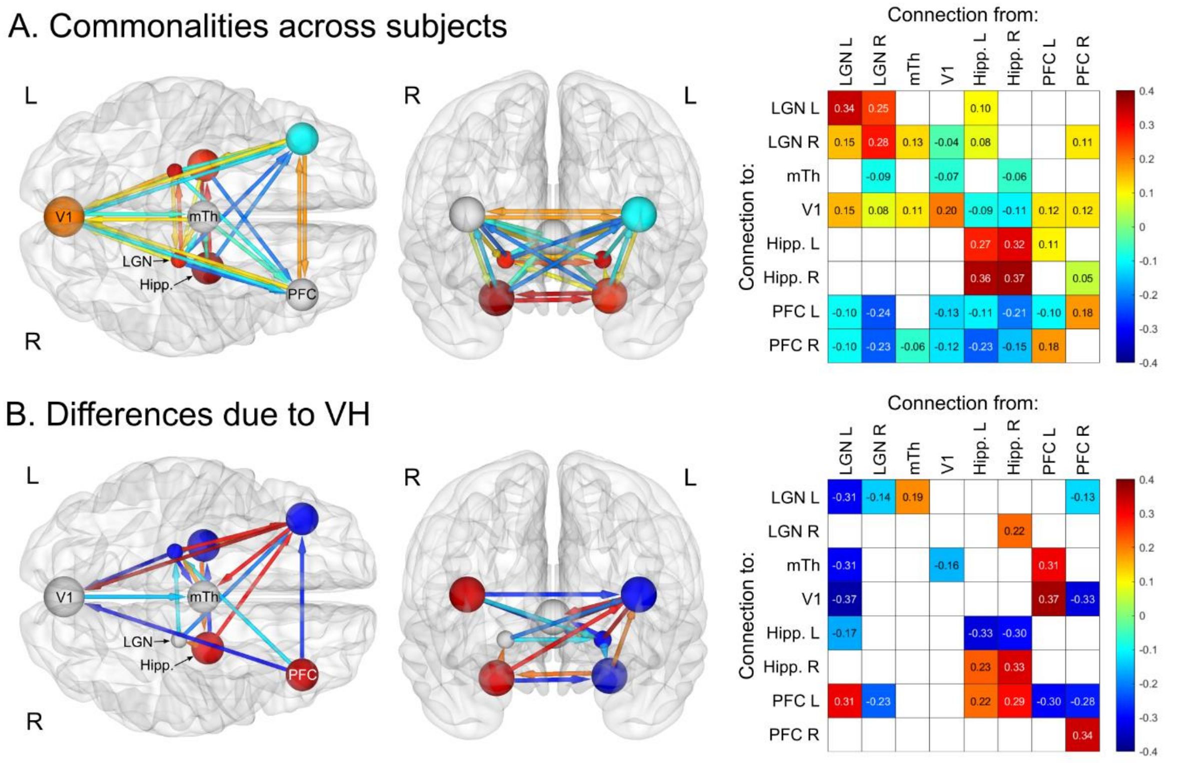

Supplementary figure 2 - **Bayesian model averaging across models generated by an automatic search over parameters.** Parameter values are averaged across the final set of 256 models generated by a data-driven automatic (greedy) search over parameters. All parameters are thresholded at posterior probability >95% of being present versus absent. **(A) Commonalities across all patients** (with and without hallucinations equivalent to the mean across subjects). Here, orange arrows / off-diagonal positive numbers reflect excitatory connectivity and blue arrows / off-diagonal negative numbers reflect inhibitory connectivity. Diagonal connectivity / self-connectivity is inhibitory by definition and is log-scale, hence leading diagonal positive numbers / orange spheres reflect more self-inhibition and leading diagonal negative numbers / blue spheres reflect disinhibition. **(B) Differences between hallucinators and non-hallucinators.** Here, orange arrows / off-diagonal positive numbers reflect increased connectivity in PD-VH versus PD-no-VH, whereas blue arrows / off-diagonal negative numbers reflect decreased connectivity. Leading diagonal positive numbers / orange spheres reflect increased self-inhibition in PD-VH versus PD-no-VH and leading diagonal negative numbers / blue spheres reflect increased disinhibition. LGN = Lateral geniculate nucleus; mTh = medial thalamus; Hipp. = hippocampus; PFC = prefrontal cortex.
